# Advancements in Sickle Cell Disease (SCD) Treatment: A Review of Novel Pharmacotherapies and Their Impact on Patient Outcomes

**DOI:** 10.7759/cureus.42847

**Published:** 2023-08-02

**Authors:** Salman J Khan, Syed Asjad Tauheed Zaidi, Syeda Fatima Murtaza, Muhammad Asif, Vinod Kumar

**Affiliations:** 1 Medicine, Mayo Clinic, Jacksonville, USA; 2 Hematology and Oncology, Mayo Clinic, Jacksonville, USA; 3 Medicine, Shalamar Medical and Dental College, Lahore, PAK; 4 Medicine, Allama Iqbal Medical College, Lahore, PAK; 5 Internal Medicine, Avera McKennan Hospital and University Health Center, Sioux Falls, USA

**Keywords:** sickle cell disease (scd), us food and drug administration, hemolytic crisis, vaso-occlusive crisis, crizanlizumab, l-glutamine, hydroxyurea use

## Abstract

Sickle cell disease (SCD) is a genetic hereditary blood disease that disrupts normal beta-globin production. Patients with SCD experience a broad range of symptoms ranging from anemia, pain crises, and jaundice to acute coronary syndrome and stroke. SCD has been treated with hydroxyurea since 1998. Three important pharmacotherapies have been approved by the Food and Drug Administration (FDA) in the past few years. L-Glutamine has shown efficacy in reducing vaso-occlusive pain crises and hospitalization. Crizanlizumab has also shown positive outcomes in patients with SCD. Voxelotor has been studied to be effective in improving hemolytic anemia and the quality of life in SCD patients. These drugs can be used alone or in conjunction with hydroxyurea. Trials have shown that these therapies have significant efficacy. The events of pain, hemolytic anemia, vaso-occlusive crises, and hospitalizations have been reduced by using these agents. In this editorial, we will discuss these advanced treatment options for patients with SCD.

## Editorial

Sickle cell disease (SCD) is the world's most common inherited blood disorder. It is caused by a mutation in the beta-globin gene on chromosome 11p15 disrupting normal beta-globin production. This mutation causes the formation of hemoglobin S (HbS), which is less soluble when deoxygenated, and causes the formation of sickle or crescent-shaped red blood cells (RBC). Currently, there are approximately 100,000 patients with SCD in the United States, with the highest incidences in African Americans and Hispanic Americans. SCD cases increased by 41% from 2000 to 2021, and 344,000 deaths were reported worldwide in 2021 [1]. The US Food and Drug Administration (FDA) approved hydroxyurea as the only treatment for SCD in 1998. Hydroxyurea impairs DNA synthesis by inhibiting ribonucleotide reductase enzymes, increasing fetal hemoglobin (HbF), and lowering white blood cells, all of which benefit SCD patients. However, 25%-30% of SCD patients experience a suboptimal or no response to hydroxyurea, and some experience toxic side effects such as myelosuppression and hepatotoxicity [2]. Studies have shown that more recently, FDA-approved pharmacotherapies are effective in these patients and yield better responses to vaso-occlusive pain and hemolytic crisis.

L-Glutamine, approved by the FDA in 2017, is an effective option for reducing painful episodes and hospitalization in SCD patients. It increases nicotinamide adenine dinucleotide (NAD+) in sickle red blood cells and reduces oxidative stress, which contributes to the complex pathophysiology of sickle cell. A randomized, placebo-controlled, double-blind, phase 3 trial of l-glutamine revealed 25% lower pain crises in patients taking l-glutamine than patients taking a placebo. It also showed 33% fewer hospitalization in the patients taking l-glutamine compared to a placebo. The incidence of acute chest syndrome (ACS), the leading cause of death in SCD patients, was also significantly lower in the l-glutamine group with only 8.6% of patients compared to 23.1% of patients in the placebo group. It has demonstrated efficacy in reducing acute complications of SCD in adults and children above five years of age. It comes in powder form and is taken orally twice daily. A subgroup analysis revealed that the benefits of l-glutamine therapy were independent of hydroxyurea use. Thus, it can be used as an alternative therapy in patients who are intolerant to hydroxyurea [3].

Crizanlizumab was approved by the FDA in 2019, and it is a useful therapy for SCD patients as monotherapy or in conjunction with hydroxyurea. It is a monoclonal antibody that acts on P-selectin, an adhesion molecule expressed on activated platelets and endothelial cells, and blocks its interaction with P-selectin glycoprotein ligand 1. P-selectin modulates cell-to-cell interactions that contribute to the primary pathogenetic mechanism of vaso-occlusion and sickle cell-related pain crises. A double-blind, randomized, placebo-controlled, phase 2 trial showed that high-dose crizanlizumab is efficacious to frequent and severe vaso-occlusive pain events. High-dose crizanlizumab (5.0 mg per kilogram body weight) lowered the frequency of pain crisis by 45.3% compared to a placebo. Also, the median hospitalization rate was lowered by 41.8% with high-dose crizanlizumab compared to a placebo. Crizanlizumab also has a low incidence of adverse effects. It is available in a 30-minute intravenous infusion starting biweekly and then monthly afterward. The main limitations are nonavailability in the pill form, high cost, and approval to use in patients above 16 years of age [4].

Voxelotor was given accelerated approval by the FDA in 2019 for adults and children above four years of age having significant hemolytic anemia and poor quality of life. Voxelotor inhibits HbS polymerization by binding reversibly to the hemoglobin (Hb). It stabilizes the oxygenated state of Hb, thus increasing RBC life, which reduces anemia and hemolysis in SCD patients. FDA designated voxelotor a breakthrough therapy after the trials showed a considerable increase in Hb in SCD patients. The multicenter, phase 3, double-blind, randomized, placebo-controlled trial showed a 1.1 g/dL increase in hemoglobin level from baseline to week 24 in the 1,500 mg voxelotor group compared to 0.1 g in the placebo group. It also revealed that 41% of participants of the 1,500 mg voxelotor group had an increase of at least 10 g/dL at week 24 compared to 9% in the placebo group. Voxelotor significantly increased hemoglobin levels and reduced the markers of hemolysis indicated by reduction in the indirect bilirubin level and percentage of reticulocytes. Voxelotor also decreases indirect bilirubin levels, reduces red-cell sickling and blood viscosity, and improves red-cell deformability. All these effects indicate the disease-modifying potential of voxelotor. It is available in pill form with minimal side effects [5].

These newly approved pharmacotherapies can be suitable alternatives to hydroxyurea or can be used along with it to maximize the efficacy of the treatment. Another positive aspect of these therapies is that there is no need for therapeutic monitoring, unlike hydroxyurea, in which frequent complete blood count and dose titration must be considered. The incorporation of these therapies in the routine management of SCD patients can address the issues of vaso-occlusive crisis and complications such as anemia and hemolytic crisis, leading to a better quality of life and disease outcomes.
